# Experiments and Logic

**Published:** 1884-04

**Authors:** Charles Mayr

**Affiliations:** Springfield, Mass.


### EXPERIMENTS AND LOGIC.

BY CHARLES MAYR, A. M., SPRINGFIELD, MASS.

The article of Dr. Miller in the December INDEPENDENT PRACTITIONER seems to me to illustrate very forcibly that a great experimenter is not necessarily a good logician, and that the one may be entirely separate from the other. There is probably none among us who is not forced to accept Dr. Miller as an experimenter of the first rank, but this does not protect him from having his logic and his deductions submitted to our criticisms. We even admit without questioning the results of his experiments, but we take strong exception to his logic. Dr. Miller is wrong in supposing that only the man who makes the original experiments is the one who is able to write the theory upon it. We should prefer, as far as theories are concerned, to have an impartial student sum up the results of the experiments. Most investigators in a special line of investigation are wont to consider their line as of paramount importance, and to lose sight of points gained in other directions. We thus have the dental chemists, like Dr. Niles, advocating mostly chemical views, Dr. Atkinson and other physicians preferring pathological views, etc. I do not wish to say that these gentlemen do not take into consideration other views, only their natural training tends to cause them to give preference to their special line of investigation over all others. We are all familiar with the story that the excellent astronomer, Copernicus, never in his life saw the planet Mercury, and yet his theory about the planet is perfectly correct. Thus, Keppler never measured any astronomical distances, but used those of Tycho de Brahe, and based upon them his theory, which is now fully endorsed; while the views of Tycho de Brahe, from whom Keppler got his facts, only excite ridicule in our days. Tycho de Brahe was one of those proud investigators who, in well earned fame, looked down upon the speculative vermin with contempt, but the speculative bugs ate up the grand astronomer.

I do not think that Dr. Miller is very clear in his definitions of fermentation and putrefaction. I never yet saw a clear statement from him of what *he* meant by that, and it is impossible to dispute with a man, with practically useful results, before one knows what he means by certain words. I have repeatedly pronounced a most concise definition of these terms, and I think all chemical factors are in favor of this definition. Dr. Miller will have to supplant it with something better before he will meet with general acceptance. If ptyaline is a ferment, than platinum is one too. Dr. Miller, in his article of December, said: We give an abridged statement. "If a man receives a stab in the abdomen and dies, we do not say he died of peritonitis, but from the stab." That is what I call defective logic. To go into the fine details of this point would lead us into a discussion on logic, but I would only point out this: Dr. Miller knows, as well as most other dentists, that many people recover from a *stab* in the abdomen, if the peritonitis which sets in is slight; nay, where with proper treatment peritonitis is prevented, a person as good as never dies from the stab; so to call the stab the cause of the death, and to consider the peritonitis something irrelevant, is illogical.* If we speak of causes, we only speak of *direct* antecedents and consequents; the direct antecedent of the death was peritonitis, and in all logic this has to be called the direct cause of his death. The other indirect causes are of an immense number. Indirectly, we can say, it is the knife that killed the man. If no stabbing implement had been invented, the man would not have died; or, it is the hand of the criminal who wielded the knife—if people were born without hands he could not have stabbed his victim, etc., with grace *ad infinitum.* Thus, as soon as we go to indirect causes and confound them with direct ones, our reasoning becomes defective. Dr. Miller's reasoning applied to the tooth appears equally faulty. Dr. Miller thinks that the mechanical injury, the softening of the dentine by acids generated by fermentation, is decay—analogous to the stab in his strange argument. Now, when we eat vinegar, the vinegar dissolves a small amouut of the tooth substance, and we would have decay all over our teeth according to Dr. Miller. This, no dentist will acknowledge as the decay under discussion. The decay under consideration is so specific that the mere action of acids is not sufficient to produce it.

But it would exceed the space of this paper to criticise more in this direction. Good reasoning may sometimes appear to travel faster than experiments, but this does not prove that the former is wrong; all it may prove is that the best experimenters are slow.

* To take *popular* expressions as scientific conclusive evidence, belongs to "*Yedes Kind weiss das*" logic, which needs no condemnation.

